# Complete response of mantle cell lymphoma with central nervous system involvement at diagnosis with acalabrutinib – Case report

**DOI:** 10.1002/jha2.830

**Published:** 2023-12-26

**Authors:** Aisling Barrett, Toby A. Eyre, Shaheel Bhuva, Mahmoud Aljurf, Riad El Fakih, Mohammad A. Ashshi, Alfadel Alshaibani

**Affiliations:** ^1^ Department of Clinical Haematology Oxford Cancer and Haematology Centre Oxford University Hospitals NHS Foundation Trust Oxford UK; ^2^ Department of Clinical Radiology Oxford University Hospitals NHS Foundation Trust Oxford UK; ^3^ Department of Stem Cell Transplant and Cellular Therapy King Faisal Specialist Hospital and Research Center Riyadh Saudi Arabia; ^4^ College of Medicine Umm Al‐Qura University Makkah Saudi Arabia

**Keywords:** acalabrutinib, case report, central nervous system lymphoma, mantle cell lymphoma, PET in lymphoma

## Abstract

Central nervous system (CNS) involvement by mantle cell lymphoma (MCL) is rare and portends a poor prognosis. We describe the first patient to have a complete response with front‐line treatment with single‐agent acalabrutinib for MCL CNS.

## Graphical abstract

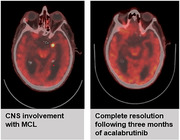

## 1 INTRODUCTION

Central nervous system (CNS) involvement by mantle cell lymphoma (MCL) is rare, observed in < 5% of cases at relapse and in < 1% at diagnosis [1]. Outcomes are poor with median overall survival (OS) as low as 3.7 months from presentation [1]. Treatment outcomes with high‐dose chemotherapy which penetrates the blood‐brain barrier (BBB) are unsatisfactory and novel therapies with different therapeutic mechanisms are required.

## 2 DISCUSSION

An 89‐year‐old male presented to King Faisal Specialist Hospital and Research Centre with acute onset of confusion and headache alongside a 3‐month history of weight loss and generalized lymphadenopathy. He had multiple comorbidities including ischaemic heart disease with coronary artery stenting performed 3 years prior, dyslipidemia, hypertension, type II diabetes mellitus, and chronic kidney disease with a baseline estimated glomerular filtration rate (eGFR) of 45 mL/min. Full blood count (FBC) at presentation revealed a hemoglobin of 8.2 g/dL but with a normal platelet and white cell count.

**FIGURE 1:**
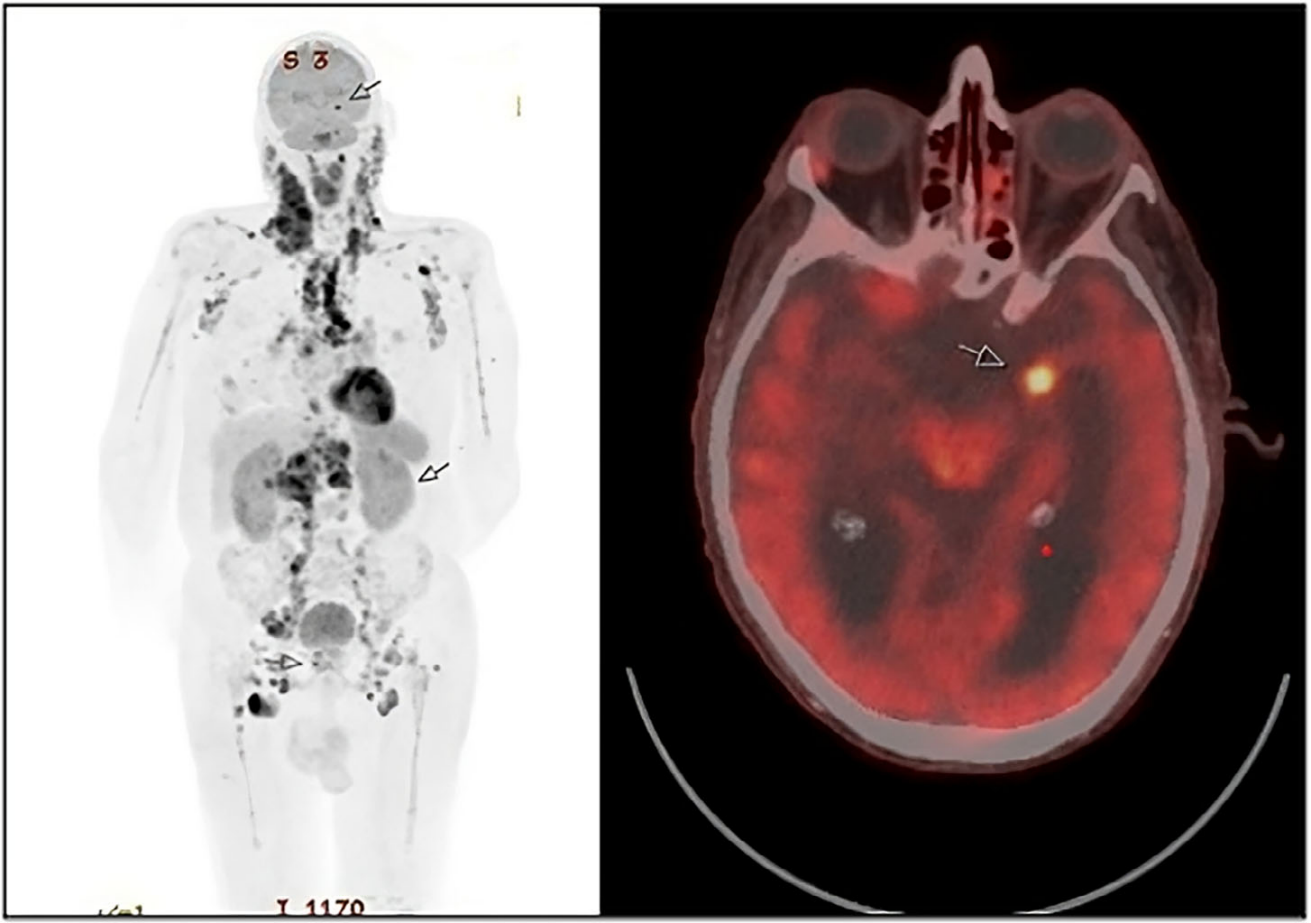
Positron emission tomography (PET) at diagnosis. This shows metabolically active lymphoma above and below the diaphragm with a focal increase in fluorodeoxyglucose (FDG)‐avidity in the medial aspect of the left temporal lobe with a standardized uptake value (SUV) max of 8.4 consistent with central nervous system (CNS) involvement by mantle cell lymphoma (MCL).

A magnetic resonance imaging (MRI) scan of the brain showed thickening of the left amygdala associated with abnormal enhancement. A positron emission tomography (PET) scan showed fluorodeoxyglucose (FDG)‐ avid lymph nodes above and below the diaphragm, and a focal increase in FDG‐ avidity in the medial aspect of the left temporal lobe with a standardized uptake value max of 8.4 consistent with the intracranial spread of lymphoma (Figure 1). A subsequent lymph node biopsy showed a CD20, SOX11, and cyclin D1‐positive pleomorphic lymphoid infiltration with a Ki67 of 50% which was negative for CD10 and BCL6, consistent with MCL. TP53 analysis was not undertaken and fluorescence in situ hybridization for CCND1 rearrangement failed. Lumbar puncture was not performed due to the increased risk of bleeding at this time due to concomitant aspirin use.

The patient became critically unwell during this admission, with the development of new atrial fibrillation and a non‐ST elevation myocardial infarction requiring management in the intensive care unit. These were managed conservatively as he was deemed unfit for percutaneous coronary intervention due to an acute kidney injury (eGFR 12 mL/min).

Conventional chemotherapy was considered inappropriate due to the patient's background comorbidities and current clinical state. Initial steroid debulking therapy with 30 mg of oral prednisolone daily for 8 days and subsequent anti‐lymphoma therapy with off‐label acalabrutinib monotherapy was commenced. Therapy was well tolerated with a transient skin rash which spontaneously resolved.

**FIGURE 2:**
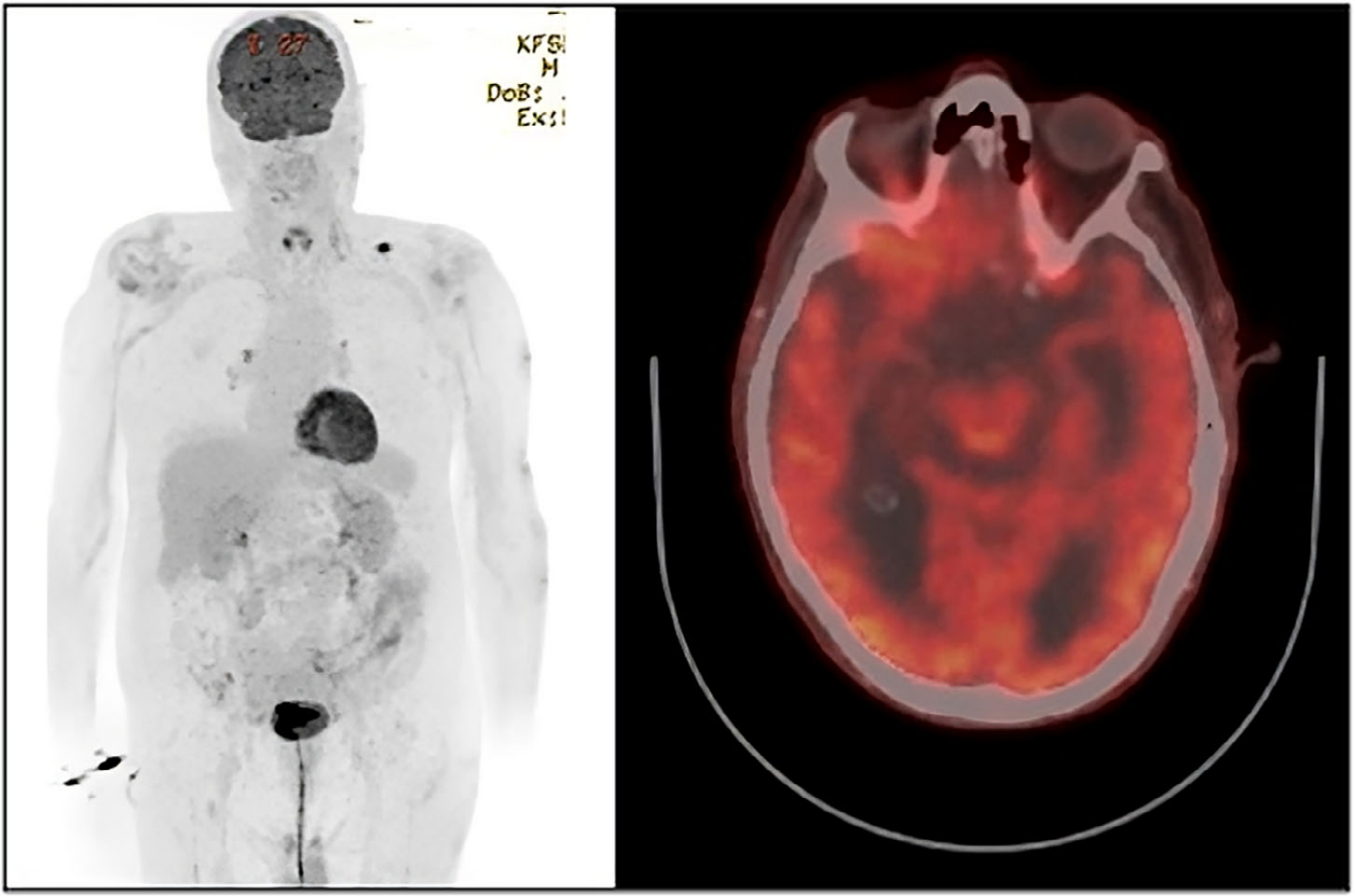
Positron emission tomography (PET) 3 months after commencement of acalabrutinib. This shows complete resolution of intracranial changes and almost complete resolution of fluorodeoxyglucose (FDG)‐avid lymphadenopathy.

A 3‐month PET scan showed an excellent partial metabolic response to acalabrutinib, with a complete response (CR) of the intracranial FDG avidity (Figure 2). A repeat MRI of the brain at 3 months also showed complete resolution of the previous abnormal findings. Treatment continued to be well tolerated and the patient has remained an outpatient in the 6 months following his admission with complete regain of cognitive function and full symptomatic resolution.

Acalabrutinib is a highly selective second‐generation covalent Bruton tyrosine kinase inhibitor (BTKi), with an improved cardiac and non‐cardiac adverse event profile in comparison to the first‐in‐class agent ibrutinib [2]. It is Food and Drug Administration‐approved for the treatment of relapsed or refractory MCL on the basis of the phase II trial ACE‐LY‐004, which demonstrated an overall response rate (ORR) of 81% in this setting with a median duration of response of 26 months [3].

CNS involvement by MCL is more common in patients with advanced‐stage disease, blastoid histological variant, and aggressive biological features [4]. Median OS from diagnosis of CNS disease either at initial diagnosis or at relapse strikingly varies in analyses, from 3.7 months in a historical cohort [1] to 50.3 months in the aforementioned study where 11% of patients were treated with a BTK inhibitor. The first‐generation BTKi ibrutinib has been shown to improve OS in CNS relapse of MCL, with a median OS of 16.8 versus 4.4 months (*p* = 0.007) in a large retrospective study of 88 patients treated with either ibrutinib or standard BBB penetrating chemotherapy [5]; randomised trials have not been performed.

Data on acalabrutinib use for CNS MCL are lacking. Acalabrutinib is pharmacologically expected to cross the BBB similarly to ibrutinib, and an upcoming phase II study will examine its use in relapsed primary CNS lymphoma [6]. There are only two recorded cases of acalabrutinib use in CNS involvement of MCL at relapse in the literature, with one patient achieving a partial response (PR) to treatment [4] and a second patient who was intolerant of ibrutinib achieving a CR with acalabrutinib [7].

## 3 CONCLUSION

To the best of our knowledge, this is the first case where acalabrutinib has been used as first‐line therapy in MCL presenting initially with CNS involvement. Our patient was unfit for a more standard intensive approach with cytarabine‐containing chemoimmunotherapy and autologous transplantation as consolidation. First‐line acalabrutinib monotherapy represents a valid treatment option where available for the management of CNS MCL in patients who are unsuitable for intensive BBB‐penetrating chemotherapy‐based approaches and may be preferable to ibrutinib in light of the well‐described improvement in toxicity profile.

## AUTHOR CONTRIBUTIONS

Aisling Barrett and Toby A. Eyre co‐wrote the manuscript. Shaheel Bhuva reviewed the radiological images for the manuscript. Mahmoud Aljurf, Riad El Fakih, Mohammad A. Ashshi, and Alfadel Alshaibani provided the data for the manuscript and reviewed the manuscript.

## CONFLICT OF INTEREST STATEMENT

Shaheel Bhuva, Mahmoud Aljurf, Riad El Fakih, Mohammad A. Ashshi, and Alfadel Alshaibani declare no conflict of interest. Aisling Barrett has received travel support from Ionis Pharmaceuticals. Toby A. Eyre has received honorarium and/ or advisory board honorarium from Roche, Gilead, KITE, Janssen, AbbVie, AstraZeneca, LOXO Oncology, Beigene, Incyte and Secura Bio, receives research support from Gilead, Beigene and AstraZeneca, has received travel support from Gilead, Takeda, and AbbVie, and served on a trial steering committee for LOXO Oncology.

## FUNDING INFORMATION

The authors have not received funding for this manuscript.

## PATIENT CONSENT STATEMENT

The corresponding author has received written consent from the patient allowing publication of their medical data.

## ETHICS STATEMENT

This article is written in compliance with the Declaration of Helsinki, and the patient provided written consent for this case report.

## CLINICAL TRIAL REGISTRATION

The authors have confirmed clinical trial registration is not needed for this submission.

## Data Availability

Data are available on request from the corresponding author and are subject to ethical and privacy restrictions.
